# Supplementary material for: Becker muscular dystrophy mice showed site-specific decay of type IIa fibers with capillary change in skeletal muscle
Source: eLife. 2025 Mar 17;13:RP100665. doi: 10.7554/eLife.100665 (PMC11913446; doi:10.7554/eLife.100665)
Supplement: Supplementary file 1. [file elife-100665-supp1.docx]

| **Supplementary File 1.** Primers used for the RT-PCR analysis | | |
| --- | --- | --- |
| 18S rRNA | Forward | 5'–TTCAAGGCAGCCTGTGATGT–3' |
|  | Reverse | 5'–ATGGTCGATCGGCTGGATTT–3' |
|  |  |  |
| Dystrophin (exons 75-77) | Forward | 5'–AACACAAAGGACGCCTGGAA–3' |
|  | Reverse | 5'–TAACCCTGTGCTTGTGTCCT–3' |
|  |  |  |
| Utrophin | Forward | 5'–TCCCCACAGCATTCTGCATT–3' |
|  | Reverse | 5'–AGAGGGAAACGTGCTGTTGA–3' |
|  |  |  |
| Neuronal nitric oxide synthase (nNOS) | Forward | 5'–TTCAAGGCAGCCTGTGATGT–3' |
|  | Reverse | 5'–ATGGTCGATCGGCTGGATTT–3' |
|  |  |  |
| Muscle ring finger protein-1 (Murf1) | Forward | 5'–TGCAGAGTGACCAAGGAGAA–3' |
|  | Reverse | 5'–ATGGCGGTCTCCACAAGTTT–3' |
|  |  |  |
| Atrogin-1 | Forward | 5'–TGCCTGTGTGCTTACAACTG–3' |
|  | Reverse | 5'–TCAAACGCTTGCGAATCTGC–3' |
|  |  |  |
| *DMD* exon 44 | Forward | 5'– GCGATTCGACAGATCAGTTG–3' |
|  | Reverse | 5'– GCATGTTCCCAGTTTTCAGG–3' |
|  |  |  |
| *DMD* exon 45 | Forward | 5'–CTGAATGCAACTGGGGAAGA–3' |
|  | Reverse | 5'–CGCAGACTCAAGCTTCCTAA–3' |
|  |  |  |
| *DMD* exon 46 | Forward | 5'–TTGTGGCTGGAAGAAGCAGA–3' |
|  | Reverse | 5'–TGCTCATCTCCAAGTGGAGT–3' |
|  |  |  |
| *DMD* exon 47 | Forward | 5'–GCGCCAGGGAATTCTAAAAC–3' |
|  | Reverse | 5'–TTGCTCTTCTGGCCTTATGG–3' |
|  |  |  |
| *DMD* exon 48 | Forward | 5'–AGGAGAGCTTGAGGTTCACT–3' |
|  | Reverse | 5'–CTGCCTGACTTGGTTGGTTA–3' |
|  |  |  |
| *DMD* exon 49 | Forward | 5'–AACAAGCGGATGTGGAAAGG–3' |
|  | Reverse | 5'–TTCACTGGCTGAGTGCTT–3' |
|  |  |  |
| *DMD* exon 50 | Forward | 5'–TCTGAGTGGGAGGCTGTAAA–3' |
|  | Reverse | 5'–TCCTCAGCTCCCGAAGTAAA–3' |
|  |  |  |
